# Concurrent Middle Cerebral Artery and Basilar Artery Occlusions Treated With Mechanical Thrombectomy in a Patient With Active COVID-19 Infection

**DOI:** 10.7759/cureus.57623

**Published:** 2024-04-04

**Authors:** Saarang Patel, Jeffrey Treiber, Jeremiah N Johnson

**Affiliations:** 1 Arts and Sciences, Seton Hall University, South Orange, USA; 2 Neurological Surgery, Baylor College of Medicine, Houston, USA; 3 Neurosurgery, David Geffen School of Medicine, University of California Los Angeles, Los Angeles, USA

**Keywords:** thrombectomy, covid-19, vascular, neurology, neurosurgery

## Abstract

We report a rare case of acute ischemic stroke from concurrent large vessel occlusions (LVOs) and subsequent successful mechanical thrombectomy revascularization in a patient with active coronavirus disease 2019 (COVID-19) pneumonia. A 59-year-old woman presented to the emergency department after one week of intermittent chest pain, dyspnea, and diarrhea found to have COVID-19 pneumonia. On hospital day three, the patient developed acute altered mental status and hemiparesis with a National Institutes of Health Stroke Scale (NIHSS) of 22. CT with angiography demonstrated concurrent occlusions of the basilar artery and the M1 segment of the right middle cerebral artery (MCA) without intracranial hemorrhage. The patient was taken for urgent mechanical thrombectomy of the basilar artery, followed by the MCA, both of which were successful (thrombolysis in cerebral infarction (TICI) 3 and 2B) and timely. Despite early revascularization, the patient did not improve clinically with absent brainstem reflexes and a full MCA territorial infarct on imaging. This case describes a rare stroke syndrome of concurrent LVOs with rapid infarct progression despite timely revascularization. This example illustrates a severe cerebrovascular complication of active COVID-19 infection and the importance of vigilance regarding stroke prevention and neurological examination monitoring.

## Introduction

Coronavirus disease 2019 (COVID-19) is a worldwide pandemic caused by the severe acute respiratory syndrome coronavirus 2 (SARS-CoV-2). This virus has infected over 774 million individuals with more than seven million deaths noted worldwide as of February 2024 [1]. Initially classified as a respiratory illness, we now know that this infection can lead to multisystem organ dysfunction because of direct and indirect effects, which include the development of arterial and venous thrombi [2-3]. Some patients with active COVID-19 infection enter a hypercoagulable state [4]. Although there are likely multiple complementary pathways leading to hypercoagulability, a core component of the hypothesis is that response to the virus activates the coagulation cascade in some individuals, which can result in pulmonary emboli, myocardial infarction, and stroke [4-7].

In this case report, we present an unusual case of acute ischemic stroke with a concurrent middle cerebral artery and basilar artery occlusion in a middle-aged female with active COVID-19 infection. We discuss her presentation, the location of the occlusions in association with underlying atherosclerotic disease, and her postintervention outcome.

## Case presentation

History and examination

**Figure 1:**
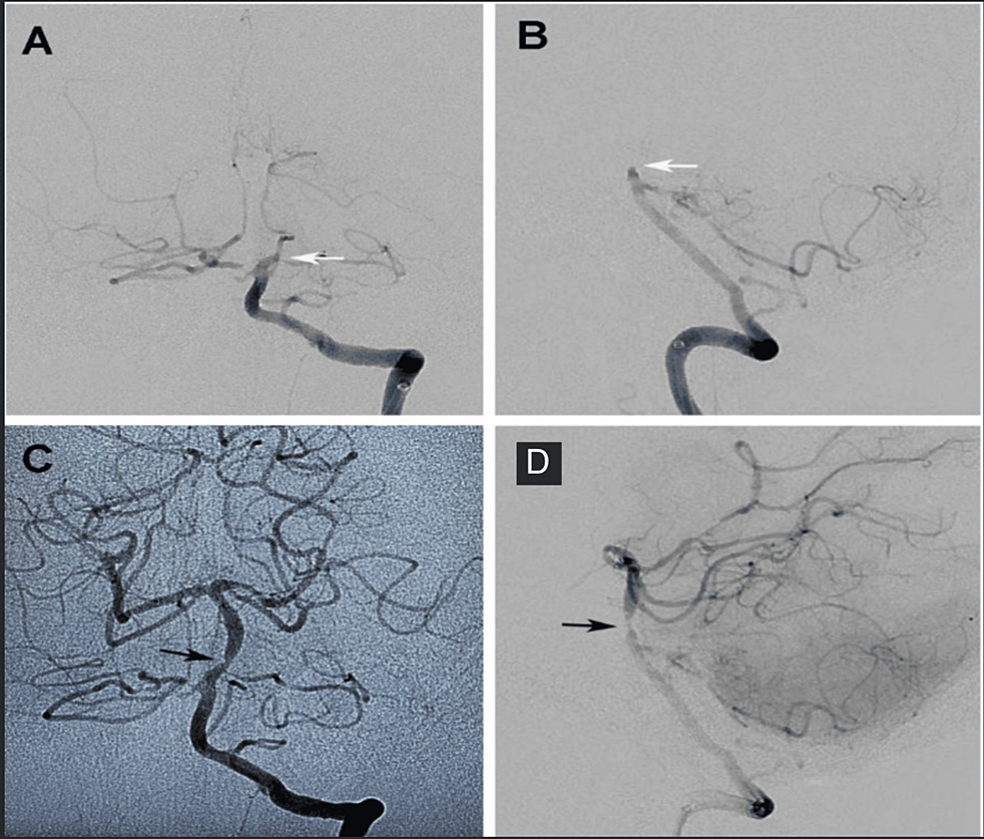
Basilar Occlusion and Revascularization Pre-thrombectomy left vertebral artery injection cerebral digital subtraction angiogram with A) AP and B) lateral views demonstrating the mid-basilar artery occlusion (white arrows). C,D) Post-thrombectomy, there is complete revascularization but notable underlying stenosis suggestive of underlying vessel wall atherosclerotic plaque (black arrows) is present.

**Figure 2:**
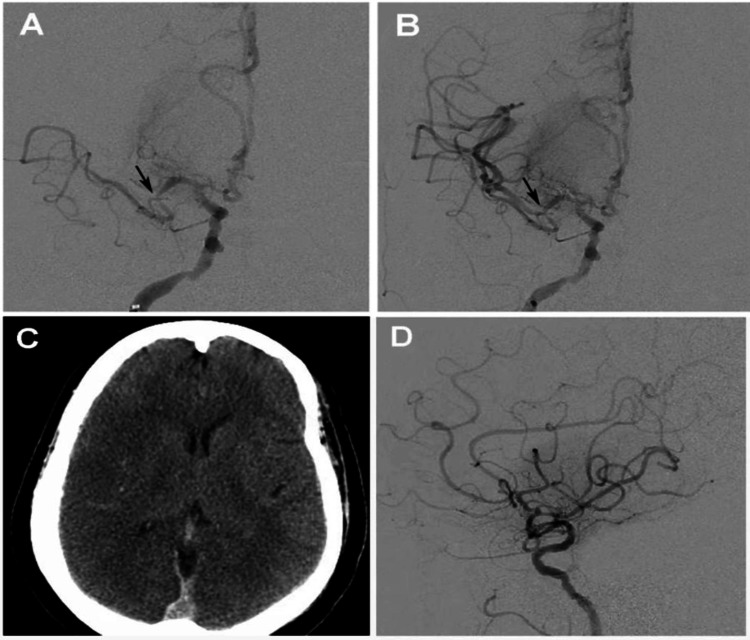
Middle Cerebral Artery Occlusion and Revascularization A) Pre-thrombectomy right internal carotid artery cerebral digital subtraction angiography (DSA) angiogram demonstrating a cutoff at the middle cerebral artery (MCA) bifurcation (black arrow) with only the anterior temporal artery filling distally. B) Post-thrombectomy, AP view shows successful revascularization of the MCA territory but notable moderate focal stenosis at the prior occlusion point (black arrow) suggestive of underlying atherosclerotic disease. C) CT brain without contrast performed 48 hours after intervention demonstrating hypodense right MCA territory cortex with loss of grey-white differentiation and right MCA territory hypodensity (edema) consistent with large volume MCA territory infarct. D) Lateral view after revascularization demonstrating flow in the superior and inferior division but delay in filling distally compared to anterior cerebral artery (ACA) territory felt to be related to infarct core.

The patient was a functionally independent 59-year-old female with a history of atrial fibrillation on apixaban, coronary artery disease status post-coronary artery bypass graft two years prior, diabetes mellitus type 2, hyperlipidemia, chronic kidney disease grade IV, and past ischemic stroke with right carotid stent placement three years prior, who had been off from all medications for one year after losing insurance coverage. She presented to our Emergency Department with one week of intermittent chest pain, dyspnea, and diarrhea. On her initial evaluation, she was hemodynamically stable and had expiratory wheezing but was not in respiratory distress or required supplemental oxygen. Chest x-ray was consistent with viral pneumonia and laboratory studies revealed a positive SARS-CoV-2 polymerase chain reaction (PCR), elevated serum troponin (0.23 ng/mL; ref 0.00-0.03 ng/mL), and acute on chronic renal failure. EKG was unremarkable, and she was started on aspirin, Plavix®, and a heparin drip because of concern for coronary ischemia. She was then admitted to the floor under a hospitalist service. Intravenous heparin was started empirically for NSTEMI, although it was discontinued after 24 hours because of low suspicion of coronary thrombus. No abnormalities in her platelet count or coagulation markers were noted. Her COVID-19 pneumonia, however, worsened over the first 24 hours, and she started requiring supplemental oxygen by nasal cannula. Intravenous dexamethasone (6mg daily) and remdesivir were started. On the third day of her hospitalization, she developed acute hypoxic respiratory failure, altered mental status, and a left-sided hemiparesis (NIHSS 22). A stroke alert was called, and she was emergently intubated, and a CT head and CT angiogram of the head and neck were performed three hours after last known to be well and demonstrated an occlusion of the M1 segment of the right middle cerebral artery and an occlusion of the mid-basilar artery with distal reconstitution via bilateral posterior communicating arteries. No intracranial hemorrhage was seen. The patient was administered IV tPA (0.09 mg/kg bolus, followed by 0.81 mg/kg infusion over one hour) and taken for emergent mechanical thrombectomy. Digital subtraction angiography confirmed persistent right MCA distal M1 segment occlusion and a basilar artery occlusion (Figures 1-2).

Intervention

To avoid brainstem ischemia, basilar artery revascularization was performed first. After femoral access, an AXS 6F Infinity guide catheter (Stryker Neurovascular Fremont, CA) was stationed in the left subclavian artery. The intracranial left vertebral artery was accessed using a Catalyst 6 (Stryker Neurovascular Fremont, CA) intermediate catheter advanced over a Velocity® microcatheter (Penumbra Alameda, CA) over a microwire. A Trevo NXT 4 mm x 41 mm Stentriever (Stryker Neurovascular Fremont, CA) was deployed across the thrombus spanning from the right posterior cerebral artery P1 segment to the proximal Basilar artery. Catalyst 6 was advanced to the face of the thrombus and aspiration was performed via Penumbra ENGINE (Alameda, CA) through Catalyst 6 for two minutes. The Stentriever and Catalyst 6 were then retracted together into the Infinity guide catheter and follow-up angiograms demonstrated a complete TICI 3 revascularizations on the first pass. The right MCA was revascularized with the same technique achieving TICI 2B revascularization with both vessels revascularized by 50 minutes total after groin puncture (4.5 hours) and three hours after the patient was last seen well. Both vessels were noted to have modest atherosclerotic narrowings at the area of occlusion (Figure 2). Revascularization of the basilar artery was achieved at 5.5 hours, with revascularization of the right middle cerebral artery being achieved at 5.5 hours.

Postoperative course

Despite successful revascularization, the patient demonstrated a poor post-procedure neurological exam with multiple absent brainstem reflexes and a dilated nonreactive pupil. A computerized tomography brain scan at 24 hours post-procedure demonstrated a large right MCA territory ischemic infarct and left cerebellar hemisphere ischemic changes (Figure 2). On post-procedure day one, the patient was comatose with eyes tonically open and an intact cough reflex. She did not have a corneal or gag reflex. Unfortunately, the unilateral pupillary dilation was not well documented, so laterality is not known. On post-procedure day two, the patient regained her lower cranial nerve function but remained comatose now with bilaterally large nonreactive pupils. At this point, the family elected to transition to comfort care and terminally extubate the patient. The patient subsequently expired.

## Discussion

In this case report, we present a middle-aged patient with concurrent large vessel occlusions (LVOs) of the basilar artery and middle cerebral artery in the setting of symptomatic COVID-19 pneumonia. The development of thrombotic complications has been associated with worsened prognosis [7]. Among the thrombotic complications of COVID-19, stroke carries the highest morbidity [8]. Observational studies and meta-analyses noted an increased incidence of stroke in COVID-19 patients (1.1-1.6%) when compared to the general population [9]. Among disease-associated conditions, stroke was found to be an independent risk factor for poorer prognosis in patients with COVID-19 [9]. Moreover, the presence of stroke within SARS-CoV-2-infected patients has been noted to have its own unique characteristics. Most notably, younger patients (<55 years old) without the presence of classic vascular risk factors have had a much larger increased incidence of stroke with COVID-19 infection than would otherwise be seen in the general population [10-12]. A prevalence of both cryptogenic stroke and large vessel stroke has been reported, even in patients with mild SARS-CoV-2 and in younger patients [10,12-17].

Simultaneous occlusions of multiple large cerebral vessels are a rare occurrence, typically seen only in cases of profound hypercoagulable and thrombophilic states [18]. Although the patient’s acute renal failure and cardiac ischemia may have been because of hypoperfusion from small to medium vessel thrombosis, platelet counts, and coagulation studies, were not suggestive of an overt hypercoagulable or thrombophilic state.

As is seen across all types of strokes, COVID-19 infection is associated with poorer prognosis in patients with an LVO [19]. A study by Ntaios et al. found that through their propensity score-matched analysis of COVID-19 patients compared to non-COVID-19 patients registered in the Acute Stroke Registry and Analysis of Lausanne Registry between 2003 and 2019, the median National Institutes of Health Stroke scale score was found to be higher in patients with COVID-19 compared to patients without COVID-19 (10 (interquartile range (IQR): 4-18) and six (IQR: 3-14), p = 0.03) [20]. Furthermore, in the 1:1 matched sample of 336 patients, 48 (27.6%) had died either as a result of their stroke (n = 26) or their COVID-19 infection (n = 22) [20]. Finally, in the 330 sample size propensity score-matched population, patients diagnosed with the COVID-19 infection were at a higher risk of severe disability (median modified Rankin scale (mRS) 4 (IQR: 2-6) and 2 (IQR: 1-4), P < 0.001), as well as death (odds ratio: 4.3, 95% CI: 2.22-8.30) [20]. Thus, as Ntaios et al. state, as well as noted in other studies, COVID-19 infection has a direct correlation with poorer outcomes in patients with stroke [20,21]. Interestingly, this held true in patients with minimally symptomatic to asymptomatic SARS-CoV-2 infection at stroke onset as well [22]. Even when both early intravenous thrombolysis and successful mechanical thrombectomy were paired together, outcomes noted in LVO patients with COVID-19 infection following stroke were poorer [22].

As with other thrombotic complications of COVID-19, the etiology of stroke in COVID-19 is believed to be due to the viral infection of endothelial cells [23]. The infection of endothelial cells in turn results in thrombus formation, as well as impacting the balance of vascular tone, which results in both diminished blood flow and ischemia [23]. The impact of platelets was also noted to play a role in stroke occurrence within patients with COVID-19 where platelet counts >450 × 109/L were predictive of thrombotic events and platelet counts below 450 × 109/L were associated with hemorrhagic events [23]. Although our patient did not have abnormalities in her platelet count or coagulation markers, her prothrombotic state was clinically apparent with the simultaneous development of two distant LVOs. Interestingly, on angiography, our patient had notable atherosclerotic disease near both occlusion sites without severe or ruptured appearing plaques. This appearance might suggest de novo clot formation from a hypercoagulable state rather than traditional thrombo-embolic mechanism as would be expected with her chronic atrial fibrillation or her extensive vascular disease. Through a common mechanism, COVID-19-related hypercoagulability may have contributed to the development of her cardiac ischemia seen on admission. While extremely rare in the general population, multivessel occlusions are being more commonly seen in COVID-19 patients, supporting the idea that de novo clot formation may serve as one mechanism of stroke attributable to the SARS-CoV-2 virus [24].

## Conclusions

We report an unusual case of simultaneous intracranial LVOs with different vascular territories in a patient with active COVID-19 infection. Unfortunately, despite a timely and successful revascularization, the patient developed a catastrophic neurological injury. This case illustrates an example of the severity of acute ischemic stroke that can be associated with COVID-19 infection, as well as the trend towards poorer outcomes in these patients despite successful revascularization. We hope that this example demonstrates the importance for medical professionals to have a high suspicion of stroke in what is typically thought of as a primarily respiratory disease, and to continue vigilance with stroke prevention in this challenging population.
